# Controversies in upper gastrointestinal oncology: neoadjuvant chemoradiotherapy versus neoadjuvant chemotherapy in oesophageal adenocarcinoma

**DOI:** 10.1016/j.esmoop.2022.100613

**Published:** 2022-11-07

**Authors:** A. Petrillo, S. Lorenzen, H.W.M. van Laarhoven

**Affiliations:** 1 Medical Oncology Unit, Ospedale del mare, Naples, Italy; 2 Department of Hematology and Oncology, Technical University, Munich, Germany; 3 Department of Medical Oncology, Amsterdam University Medical Centers, Cancer Center Amsterdam, Amsterdam; 4 University of Amsterdam, Amsterdam, The Netherlands

**Click here to listen to the Podcast**

Recent data have presented intriguing information about the treatment of patients with oesophageal adenocarcinoma. However, the actual standard of care in the neoadjuvant treatment for nonmetastatic tumour remains one of the hottest controversial topics in upper gastrointestinal tumours.

In this podcast, Dr Angelica Petrillo interviews Prof. Hanneke WM van Laarhoven and Prof. Sylvie Lorenzen about the best choice for neoadjuvant treatment for nonmetastatic oesophageal adenocarcinoma: chemoradiotherapy or chemotherapy? The two experts debate about the pros and cons of each approach alongside future perspectives, biomarkers and implication for patients’ selection.

Prof. van Laarhoven comments in favour of neoadjuvant chemoradiotherapy in this setting, referring to the data from the CROSS trial and its update after 10 years. Those data clearly showed that neoadjuvant chemoradiotherapy improved the survival outcomes, along with good tolerability, when compared with neoadjuvant chemotherapy in patients with oesophageal adenocarcinoma even after long-term follow-up.

By contrast, Prof. Lorenzen discusses about the role of neoadjuvant chemotherapy, commenting on the data from the FLOT-4 trial, which also showed a benefit in survival in this subgroup of patients with a manageable safety profile.

They then discuss about the role of immunotherapy in this field, based on the results from the CHECKMATE 577 trial, and the use of biomarkers, such as human epidermal growth factor receptor 2 (HER2), programmed death-ligand 1 (PD-L1), circulating tumour DNA (ctDNA) and microsatellite status.

However, direct comparisons between the two debated strategies do not exist yet. Recently, the preliminary results from the NEO-AEGIS trial, which compared perioperative chemotherapy with chemoradiotherapy in this setting, were presented at the American Society of Clinical Oncology (ASCO) congress. The trial showed similar results in both arms in terms of disease-free survival. Of note, 85% of patients were treated with the insufficient MAGIC regimen epirubicin, cisplatin and fluorouracil or capecitabine (ECF/EOX) and not according to the current treatment recommendations with the docetaxel, oxaliplatin, and fluorouracil/leucovorin (FLOT) protocol. However, several phase III trials comparing head-to-head the two neoadjuvant strategies in oesophageal adenocarcinoma are ongoing; their results are awaited to provide a definitive recommendation about the best treatment choice, if any, in this setting.

